# Successful alemtuzumab retreatment in multiple sclerosis following previous diffuse alveolar haemorrhage

**DOI:** 10.1177/13524585251365791

**Published:** 2025-08-20

**Authors:** Dylan Haixiang Zhao, Gina Hadley, Marco Pisa, Peter Saunders, Gabriele De Luca

**Affiliations:** Medical Science Division, University of Oxford, Oxford, UK; Department of Clinical Neurosciences, University of Oxford, Oxford, UK; Department of Clinical Neurosciences, University of Oxford, Oxford, UK; Oxford University Hospitals Foundation Trust, Oxford, UK; Department of Clinical Neurosciences, University of Oxford, Oxford, UK

**Keywords:** Alemtuzumab, multiple sclerosis, disease-modifying therapies, relapsing /remitting

## Abstract

Alemtuzumab-induced diffuse alveolar haemorrhage (DAH) is a rare but serious complication in people with relapsing-remitting multiple sclerosis (RRMS). Evidence supporting retreatment despite adverse events remains limited. We report a 29-year-old female who developed DAH during the first alemtuzumab cycle and was subsequently retreated 1 year later following interdisciplinary advice from respiratory. There was no recurrence of DAH. This case report provides evidence towards retreatment feasibility with regular monitoring and low thresholds for laboratory and radiological investigations.

## Introduction

Alemtuzumab, an anti-CD52 monoclonal antibody,^1^ is a disease-modifying therapy (DMT) used to treat active relapsing-remitting multiple sclerosis (RRMS) in two cycles 1 year apart. It is also used in solid organ transplant and certain haematological malignancies. Alemtuzumab’s mechanism in RRMS is suggested to involve initial reduction in peripheral lymphocytes with beneficial immune cell repopulation, reducing relapse rate.^2^

Alemtuzumab has several side effects, including infusion-associated reactions and acquired-autoimmune disease.^3^ The Food and Drug Administration (FDA) has warned of serious cardiovascular side effects.

Diffuse alveolar haemorrhage (DAH) is a clinical-pathological syndrome involving red blood cell accumulation in alveolar spaces caused by microcirculatory injury. It has been reported in several cases post-alemtuzumab, including in RRMS.^4–6^ In most cases, DAH leads to discontinuation of alemtuzumab given potentially fatal consequences. Evidence regarding second-cycle retreatment thus remains limited.

We report the outcome of alemtuzumab retreatment for an RRMS patient who developed DAH during the first cycle. She was retreated successfully under enhanced clinical surveillance without recurrence.

## Case report

**Figure 1.**
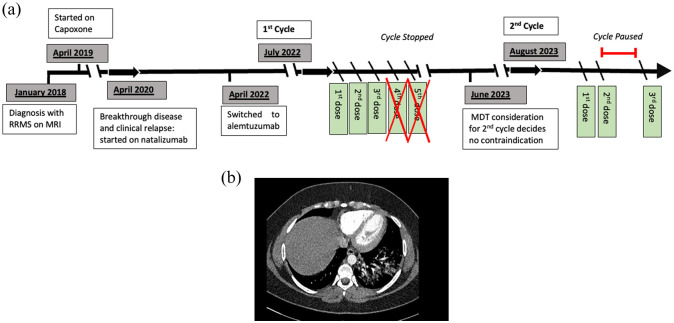
(a) Timeline of patient’s multiple sclerosis and disease-modifying treatment history. Alemtuzumab’s treatment has been written in detail highlighting clinical symptoms and omitted/suspended doses. (b) CTPA scan demonstrating pulmonary haemorrhage.

A 29-year-old female with a background history of asthma, migraine, irritable bowel syndrome, polycystic ovaries and idiopathic angioedema was diagnosed with RRMS in 2018. Following breakthrough disease activity on glatiramer acetate, she received 21 natalizumab infusions over 26 months, switching to alemtuzumab in July 2022 due to high JC virus titre^7^ (Figure 1(a)).

Alemtuzumab pre-treatment investigations were unremarkable (Supplementary Appendix 1 for pre-treatment tests). The first two infusions, pre-treated with 1 g methylprednisolone, were associated with transient mild chest tightness and discomfort mentioned by the patient just before the third infusion. Her overnight accommodation was on a farm, and she had taken corticosteroid and salbutamol for a presumed asthma flare. An electrocardiogram (ECG) demonstrated sinus tachycardia, a troponin was negative, and there was a slight wheeze on her chest. She was given a salbutamol nebuliser, and the infusion was started.

On day 4, prior to infusion, she had persistent coughing and haemoptysis described as ‘asthma with a burning sensation’. Bloods showed a markedly elevated D-dimer (18,752/L FEU; normal range = 0–500), reduced lymphocyte cell count (0.01 × 10^9^/L) and computerised tomography pulmonary angiography (CTPA) demonstrated diffuse ground glass changes which tended towards peribronchial consolidation in the left lower lobe consistent with DAH (Figure 1(b)). She was admitted for overnight observation, and the remaining two alemtuzumab infusions were not administered.

Subsequent investigations were undertaken to exclude other diagnoses, including blood work (Table 1), an echocardiogram (within normal limits) and an unsuccessful bronchoscopy attempt for bronchoalveolar lavage. Follow-up CT demonstrated complete resolution of ground glass changes 16 days after haemoptysis. A diagnosis of alemtuzumab-associated DAH was made.

**Table 1.** Investigations post DAH.

	Investigations
Bloods	ANA, ANCA, immunoglobulins, complement C3, C4, ENA myositis blot, ESR, Pro-BNP
Respiratory investigations	Spirometry with TLCO/KCO
	Bronchoscopy
	High-resolution CT chest
Cardiology investigations	Echocardiogram

Respiratory team input thought it safe to pursue a second cycle of alemtuzumab under close inpatient observation. Some literature suggests DAH can recur with rechallenge,^5^ although there is no evidence on risk reduction.

A year later, the second cycle of alemtuzumab was started with dalteparin prophylaxis due to Venous Thromboembolism (VTE) risk, under ongoing review given previous DAH. The first dose was well tolerated, but shortness of breath and chest pain emerged during the second dose similar to the first cycle. Bloods revealed a markedly elevated D-dimer (26,663 mg/L FEU) and reduced lymphocyte cell count (0.01 × 10^9^/L). A CTPA ruled out haemorrhage or other pulmonary pathology, and an ECG was within normal limits. The third dose and dalteparin prophylaxis were held, and her clinical status was closely monitored over subsequent days.

Given D-dimer normalisation, serially normal chest X-rays and clinical stability, it was deemed safe to proceed with the third dose with regular premedication after respiratory input. This was well tolerated save for transient, mild chest discomfort. She remained clinically well and was discharged. The patient has since remained clinically and radiologically stable with no respiratory or systemic sequelae.

## Discussion

We present a patient with highly active RRMS undergoing alemtuzumab treatment who developed DAH resulting in acute severe respiratory distress.^5^ Cases of DAH are limited to Pharmacovigilance Risk Assessment Committee (PRAC) and case reports in the literature.^5^

Initial differential diagnoses included vascular phenomenon, autoimmunity and infection. Given the short latency between initiation and onset, autoimmunity was unlikely, supported by a negative autoimmune screen. Rapid resolution of CT changes within 16 days precluded infection, and normal cardiovascular investigations supported the most likely diagnosis of DAH. Ideally bronchoscopy should be performed to exclude infection and confirm the presence of haemosiderin-laden macrophages: this was not tolerated.

DAH should be considered early in patients developing chest symptoms after alemtuzumab-infusion. The investigation of choice should be CTPA/CT to rule out pulmonary embolism and other complications, such as pericardial effusion, as a chest X-ray may not demonstrate abnormalities seen on CT. Importantly, the evidence for retreatment is unclear: we provide an approach to retreatment decision-making.

Literature review^5^ revealed 14 cases of DAH without other overt symptoms across all conditions treated with alemtuzumab: three received a single 30 mg dose (two renal transplant, one chronic rejection of a lung transplant), while the remaining 11 had highly active RRMS and received a low-dose treatment regimen. RRMS patients ranged between 25 and 36 years of age and developed early symptoms within days 1–4. Complete resolution of opacities was seen in 9 of the 11 RRMS patients within 2 weeks.

Previous studies advised caution for DAH-congruent symptom reoccurrence following retreatment.^5^ Mechanisms of DAH originate from damaged alveolar capillaries which may be triggered by cytokine release in some conditions.^8^ This is supported in this case by the acutely raised D-dimer in both infusions. However, factors influencing higher risk of recurrence are poorly understood. Several factors influenced our rationale to retreat. Initiation of an alternative DMT with different immune-system mechanisms may lead to unknown long-term complications and immunological interactions, especially given this patient’s previous treatment exposure. Importantly, the patient preferred retreatment with alemtuzumab given the reduced relapse rate and dose scheduling. Of course, a decision not to retreat or use another DMT risks relapse and concomitant disability accumulation. With respiratory input, more extensive pre-treatment investigations were undertaken (Table 1), and a decision to retreat on an inpatient basis with increased monitoring was made.

Although an elevation in D-dimer consequential to lymphocyte cytolysis can be seen with alemtuzumab,^9^ the increase here was an order of magnitude greater than previously observed. Respiratory consensus raised the possibility of acute cytokine release syndrome, an increase in circulating cytokines and immune cell hyperactivation that lead to an elevated D-dimer. This has been described in two cases of kidney transplant where alemtuzumab exposure resulted in flash pulmonary oedema and acute respiratory distress that recovered rapidly.^10^

## Conclusion

Alemtuzumab-induced DAH should be considered when new chest symptoms appear and is not an absolute contraindication for retreatment even with the D-dimer levels reported herein. Retreatment in these cases should involve input from respiratory for timely investigation and inpatient admission for monitoring during the second cycle. Individual cases should have risks and benefits weighed before retreatment is pursued. Consideration of flagging such rare cases to the Pneumotox collaboration (https://www.pneumotox.com/drug/index/) may help in better understanding this risk in future cases.

## Supplemental Material

sj-docx-1-msj-10.1177_13524585251365791 – Supplemental material for Successful alemtuzumab retreatment in multiple sclerosis following previous diffuse alveolar haemorrhage Supplemental material, sj-docx-1-msj-10.1177_13524585251365791 for Successful alemtuzumab retreatment in multiple sclerosis following previous diffuse alveolar haemorrhage by Dylan Haixiang Zhao, Gina Hadley, Marco Pisa, Peter Saunders and Gabriele De Luca in Multiple Sclerosis Journal

## References

[1] Gross CC Ahmetspahic D Ruck T , et al. Alemtuzumab treatment alters circulating innate immune cells in multiple sclerosis. Neurol Neuroimmunol Neuroinflamm 2016; 3: e289. 10.1212/NXI.0000000000000289 PMC5063395 27766281

[2] Cohen JA Coles AJ Arnold DL , et al. Alemtuzumab versus interferon beta 1a as first-line treatment for patients with relapsing-remitting multiple sclerosis: A randomised controlled phase 3 trial. Lancet 2012; 380: 1819–1828. 23122652 10.1016/S0140-6736(12)61769-3

[3] Havrdova E Horakova D Kovarova I . Alemtuzumab in the treatment of multiple sclerosis: Key clinical trial results and considerations for use. Ther Adv Neurol Disord 2015; 8: 31–45. 25584072 10.1177/1756285614563522 PMC4286943

[4] Myro AZ Bjerke G Zarnovicky S , et al. Diffuse alveolar hemorrhage during alemtuzumab infusion in a patient with multiple sclerosis: A case report. BMC Pharmacol Toxicol 2018; 19: 75. 30454022 10.1186/s40360-018-0267-5 PMC6245888

[5] Drop BRH Zemel D Wokke BHA , et al. Diffuse alveolar hemorrhage as an early complication of alemtuzumab treatment: A case report of a multiple sclerosis patient and an overview of 14 cases. Mult Scler Relat Disord 2021; 47: 102614. 33249378 10.1016/j.msard.2020.102614

[6] Bianco A Mari P-V Larici AR , et al. Alemtuzumab-induced lung injury in multiple sclerosis: Learning from adversity in three patients. Mult Scler Relat Disord 2020; 37: 101450. 31675637 10.1016/j.msard.2019.101450

[7] Tugemann B Berger JR . Improving risk-stratification of natalizumab-associated PML. Ann Clin Transl Neurol 2021; 8: 696–703. 33539683 10.1002/acn3.51130 PMC7951098

[8] Thomas K Eisele J Rodriguez-Leal FA , et al. Acute effects of alemtuzumab infusion in patients with active relapsing-remitting MS. Neurol Neuroimmunol Neuroinflamm 2016; 3: e228. 10.1212/NXI.0000000000000228 PMC4853056 27213173

[9] Libertinova J Meluzinova E Nema E , et al. Elevated D-dimer as an immediate response to alemtuzumab treatment. Mult Scler J 2021; 27: 151–154. 10.1177/1352458520904277 32077356

[10] Roberts DJ Olyaei A Rehman S , et al. Alemtuzumab-induced cytokine release syndrome: An under-appreciated risk in treatment of delayed renal allograft function. Trends Transp 2020; 13: 0279.

