# Supplementary material for: Successful alemtuzumab retreatment in multiple sclerosis following previous diffuse alveolar haemorrhage
Source: Mult Scler. 2025 Aug 20;31(14):1684–7. doi: 10.1177/13524585251365791 (PMC12644252; doi:10.1177/13524585251365791)
Supplement: sj-docx-1-msj-10.1177_13524585251365791 – Supplemental material for Successful alemtuzumab retreatment in multiple sclerosis following previous diffuse alveolar haemorrhage [file sj-docx-1-msj-10.1177_13524585251365791.docx]

**Supplementary Documents**

**Appendix 1**

Pre-treatment Protocol:

Listeria-free diet to be followed 6 weeks prior to infusion.

Cervical smears up to date as per national screening programme

Blood tests:

Full blood count, liver function tests, urea and electrolytes, varicella zoster virus, thyroid function, Hepatitis B and Hepatitis C virus screening, HIV screening, TB, urinalysis with microscopy.
